# Comparison of the effect of lecture and blended teaching methods on students’ learning and satisfaction

**Published:** 2014-10

**Authors:** ROYA SADEGHI, MOHAMMAD MEHDI SEDAGHAT, FARAMARZ SHA AHMADI

**Affiliations:** 1 Department of Health Education and Promotion, School of Public Health, Tehran University of Medical Sciences, Tehran, Iran;; 2 Department of Medical Entomology, School of Public Health, Tehran University of Medical Sciences, Tehran, Iran;; 3 Department of Health Education and Health Promotion, School of Public Health, Tehran University of Medical Sciences, Tehran, Iran

**Keywords:** Learning, Lecture, Tuberculosis

## Abstract

**Introduction: **Blended learning, a new approach in educational planning, is defined as an applying more than one method, strategy, technique or media in education. Todays, due to the development of infrastructure of Internet networks and the access of most of the students, the Internet can be utilized along with traditional and conventional methods of training. The aim of this study was to compare the students’ learning and satisfaction in combination of lecture and e-learning with conventional lecture methods.

**Methods: **This quasi-experimental study is conducted among the sophomore students of Public Health School, Tehran University of Medical Science in 2012-2013. Four classes of the school are randomly selected and are divided into two groups. Education in two classes (45 students) was in the form of lecture method and in the other two classes (48 students) was blended method with e-Learning and lecture methods. The students’ knowledge about tuberculosis in two groups was collected and measured by using pre and post-test. This step has been done by sending self-reported electronic questionnaires to the students' email addresses through Google Document software. At the end of educational programs, students' satisfaction and comments about two methods were also collected by questionnaires. Statistical tests such as descriptive methods, paired t-test, independent t-test and ANOVA were done through the SPSS 14 software, and p≤0.05 was considered as significant difference.

**Results: **The mean scores of the lecture and blended groups were 13.18±1.37 and 13.35±1.36, respectively; the difference between the pre-test scores of the two groups was not statistically significant (p=0.535). Knowledge scores increased in both groups after training, and the mean and standard deviation of knowledge scores of the lectures and combined groups were 16.51±0.69 and 16.18±1.06, respectively. The difference between the post-test scores of the two groups was not statistically significant (p=0.112). Students’ satisfaction in blended learning method was higher than lecture method.

**Conclusion: **The results revealed that the blended method is effective in increasing the students' learning rate. E-learning can be used to teach some courses and might be considered as economic aspects. Since in universities of medical sciences in the country, the majority of students have access to the Internet and email address, using e-learning could be used as a supplement to traditional teaching methods or sometimes as educational alternative method because this method of teaching increases the students’ knowledge, satisfaction and attention.

## Introduction

Training is known as an agent of change and progress in human. Improvement of educational quality has been considered in medical fields, and its importance is growing gradually (1). Most psychologists believe that transmitting the Education to Learners is related to educational conditions, and this education should be organized for each learner based on her/his talent and capability (2).

One of the common methods that have a special place in the training programs is lecture. Lecture is a simple, fast and cheap method to present the vast issues to a lot of groups of learners (3). Inactiveness of the students, tiring long lectures, one-way communication, and fast forgetting of the issues are the disadvantages of this method (4). According to studies, in lecture method about 80% of presented trainings are forgotten within 8 weeks (5).

Due to the limitations of the lecture method, many experts emphasize completion of traditional teaching methods and use of blended teaching methods. The blended education is actually a combination of two or more methods that use other teaching methods such as multimedia courses, seminars and e-learning, in addition to the presence classes (6). Recent studies suggest that a combination of face to face training with e-learning education method is more flexible than other methods (7).

E-Learning refers to the educational system in which educator and trainees are separated by physical distance but with the help of technology, equipment and tools they are linked together (8). E-Learning has its own limitations; for example, it cannot be replaced with the teacher, human interaction and communication in face to face classroom (9).

Several studies have been conducted at several universities in the country to compare the traditional methods with blended techniques of teaching, and different results are obtained. In this study, we aimed to compare the learning outcomes and student satisfaction in lecture method with blended method (lecture and e-learning) in Tehran University of Medical Sciences School. 

## Methods

This quasi-experimental study was conducted on the sophomore students of public health school, Tehran University of Medical Sciences in 2012-2013. Four classes of the school were randomly selected and divided into two groups. Education in two classes (45 students) was in the form of lecture method and in the two others (48 students) blended method was used (e-Learning and lecture methods).

Educational materials about TB disease in two classes (45 students) were presented as a lecture to the students by teacher during two 45-minute sessions. Educational contents were simply presented through PowerPoint slides at each session. In the other group (48 students), educational materials were presented as a combination of lectures and e-learning method. In this group, in addition to teaching the content through lecture, other materials were sent to students in 6 messages via e-mail. Also, the educational contents were placed on a blog that was designed by the researchers. Students were able to share their comments and questions at the forum with the instructor and other students.

Teaching of both groups was performed by the same instructor, and educational content was similar in both groups. Educational content included the epidemiology of tuberculosis, transmission, symptoms, treatment, and prevention methods. The aims of the study, procedure and instructional purposes were explained to the students.

The questionnaire used included demographic information, 20 questions about TB disease, and also 6 questions about student’s satisfaction about the training method. Because all of the participants in this study had e-mail address, the questionnaires were sent via e-mail to them. Pre-test and post-test were taken by electronic questionnaire designed by the researchers. E-questionnaire was designed using Google Document software. So, the questions on this application were prepared and then sent to the students’ e-mail. After the students answered the questions and pressed the OK button, the completed questionnaires were automatically sent to the researcher. The validity of the questionnaire was determined by four faculty members and its reliability was confirmed using Kuder-Richardson (r=0.80). Data analysis was done in SPSS 14 using paired t-test, independent t-test, ANOVA and descriptive statistics. A p≤0.05 was considered as significant. 

## Results

The total number of included samples in the study was 93 sophomore students of public health school, Tehran University of Medical Sciences, whose mean age was 20.28±1.54. There were not statistically significant differences between demographic variables in the two groups (p>0.05).

Initial assessment of the students' knowledge about TB showed that 43 students (95.5%) in the lecture group and 47 (97.7%) in the blended group had average scores. None of the students in the two groups before training about TB showed a high level of Knowledge. After the training, 40 (88.9%) students in the lecture group and 44 (91.7%) in the blended group revealed a good level knowledge (Table 1).

**Table1:** Frequency of students’ knowledge about tuberculosis in the lecture and blended groups

**Knowledge**	**Lecture method**			**Blended method**		
	**Low (<10)**	**Average (11-15)**	**High (16-20)**	**Low (<10)**	**Average (11-15)**	**High (16-20)**
**Pre-test ** **(%)**	2 (4.5)	43 (95.5)	0	1 (2.1)	47 (97.9)	0
**Post-test ** **(%)**	0	5 (11.1)	40 (88.9)	0	4 (8.3)	44 (91.7)

Comparing pre and post-test mean scores of the two methods showed that post-test scores were higher in comparison with pre-test scores in both methods. The mean scores of the lecture method increased from 13.18 to 16.51 and this difference was statistically significant (p=0.001). Similarly, the mean score of blended teaching method increased from 13.35 to 16.81 and this increase was also statistically significant (p=0.001) (Table 2).

**Table 2:** The mean and standard deviation of students' knowledge in the lecture and blended groups before and after training

**Group**	**Lecture method** **(Mean±SD)**	**Blended method** **(Mean±SD)**	**Independent t-test**
**Pre-test**	13.18±1.37	13.35±1.36	t=0.623, df=91, p=0.535
**Post-test**	16.51±0.69	16.81±1.06	t=1.605, df=91, p=0.112
**Range**	3.33	3.46	
**Paired t-test**	t=13.540, df=44, p=0.001	t=13.887, df=47, p=0.001	

25 (55.5%) students in the lecture group and 34 (70.9%) in the blended teaching group were satisfied with the teaching methods received. And the level of dissatisfaction with the teaching of lectures and blended methods was 28.9% and 8.3%, respectively (Table 3).

**Table 3:** Frequency of students’ satisfaction about teaching method in the lecture and blended groups

**Group**	**Satisfaction**			**ANOVA test**
	**Satisfied**	**Partly satisfied**	**Not satisfied**	
**Lecture method (%)**	25 (55.5)	7 (15.6)	13 (28.9)	F=3.425, p=0.03
**Blended method (%)**	34 (70.9)	10 (20.8)	4 (8.3)	

## Discussion

This study compared the effect of traditional lecture method and blended teaching method on students’ learning and satisfaction. Their knowledge about tuberculosis disease increased after the training compared to before it in both groups. The difference between the scores before and after training in each group was statistically significant. In other studies, teaching led to increased knowledge scores in participants (10-12).

The study by Khatooni showed that education in both lecture method and e-learning method increased the nurses' knowledge about the influenza disease (13). This increased knowledge in both lecture and blended methods showed the effectiveness of educational programs to raise the students’ knowledge.

Many studies have been done with the aim of comparing traditional methods of teaching with those active and learner-centered methods, and different results are obtained. In some studies, the participants' scores were not different between the two groups (14-17).

Jafari compared the effects of teaching on students' learning in medical biochemistry course, and showed that the differences between lecture and blended teaching methods on students’ learning were not significant (18). Hugenholtz also showed that both e-learning and traditional methods have been effective in raising the learners’ knowledge, and there was no significant difference between them (19).

In some studies aiming to increase the students’ knowledge of, lecture method was better than active and learner-centered methods. Namnabati et al. compared lecture and problem-based learning methods in nursing students. Their results showed that learning scores and retention rates in lecture method were more effective in nursing students than problem-based learning method (20). Also Javid’s study showed that the effect of the learning rate in lecture method was more than the problem-based method (21).

In some studies, the blended teaching and learner-centered methods showed better results in comparison with traditional lecture method (22-24). Hassanpour et al. in a study compared two lecture and problem-based methods on nursing students, and showed that problem-based method had a greater impact on students’ attitude, behavior and learning (25). Bahadorani et al. compared three online training, face to face and blended methods on medical students and showed the scores of the learners' knowledge and skills in blended teaching method were higher than those in the two other methods (26).

In this study, results also showed that the scores of students’ knowledge in blended teaching method were higher than the lecture method, but this difference was not statistically significant. These differences in the obtained results from the comparison of educational methods could have several reasons such as differences in learners, instructors, and how the curriculum is designed. So it is important to consider the circumstances, resources and training objectives of the educational program. According to this study, both methods increase the participants' knowledge, but students were more satisfied in blended teaching method. In some studies, the students’ satisfaction in learner-centered and blended methods was greater than that in the lecture method (14, 18, 27). The study of Norozi et al. showed that the students’ satisfaction in blended teaching method was greater than that in the traditional lecture method (4). Students in blended teaching method are confronted with a new kind of education method that leads to more incentive, participation and satisfaction. In this study, e-learning was chosen as a supplement of the lecture method. Besides raising the students’ knowledge, their other skills will be challenged. Students will be encouraged actively to participate in achievement of the required knowledge by using the Internet and E-learning, and students are more satisfied with this method in comparison with the blended method. The positive aspect of this study is the use of modern teaching methods by applying E-questionnaire as a useful and efficient tool. So other researchers can use E-questionnaire for collecting data, because the advantages of this tool include time and cost-effectiveness.

## Conclusions:

Both lecture and blended methods significantly raise the students’ knowledge. Because the students’ satisfaction and cost effectiveness in the blended method were more than lecture method, we suggest that the teachers should use e-learning as a complementary approach to combine theoretical teaching methods. 
